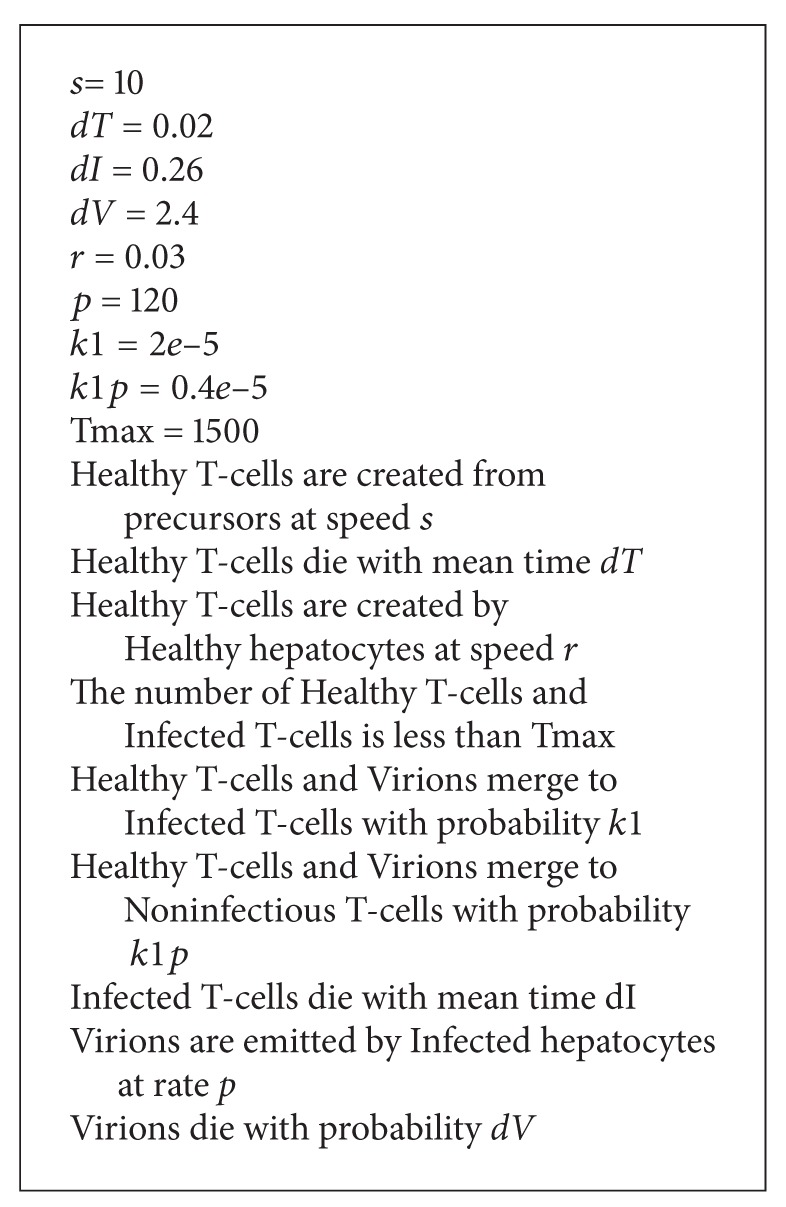# ModeLang: A New Approach for Experts-Friendly Viral Infections Modeling

**DOI:** 10.1155/2013/320715

**Published:** 2013-12-18

**Authors:** Szymon Wasik, Tomasz Prejzendanc, Jacek Blazewicz

**Affiliations:** ^1^Institute of Computing Science, Poznan University of Technology, Piotrowo 2, 60-965 Poznan, Poland; ^2^Institute of Bioorganic Chemistry, Polish Academy of Sciences, Z. Noskowskiego 12/14, 61-704 Poznan, Poland

## Abstract

Computational modeling is an important element of systems biology. One of its important applications is modeling complex, dynamical, and biological systems, including viral infections. This type of modeling usually requires close cooperation between biologists and mathematicians. However, such cooperation often faces communication problems because biologists do not have sufficient knowledge to understand mathematical description of the models, and mathematicians do not have sufficient knowledge to define and verify these models. In many areas of systems biology, this problem has already been solved; however, in some of these areas there are still certain problematic aspects. The goal of the presented research was to facilitate this cooperation by designing seminatural formal language for describing viral infection models that will be easy to understand for biologists and easy to use by mathematicians and computer scientists. The ModeLang language was designed in cooperation with biologists and its computer implementation was prepared. Tests proved that it can be successfully used to describe commonly used viral infection models and then to simulate and verify them. As a result, it can make cooperation between biologists and mathematicians modeling viral infections much easier, speeding up computational verification of formulated hypotheses.

## 1. Introduction

Computational modeling in biology is a new rapidly developing discipline. It connects two different groups of disciplines, that is, computational mathematics and computer science with biology, with mutual benefits. Mathematicians and computer scientists help to process large amounts of data and to find regularities in them, and in turn biology provides challenging problems that are not found in other disciplines [1]. However, at the point of contact of such different disciplines, communication problems arise. Biologists often have insufficient knowledge of mathematics and computer science to describe biological systems in a formal way and when mathematicians and computer scientists do this on their behalf, biologists have difficulties understanding and verifying such descriptions. Moreover, mathematicians themselves have problems gathering coherent, precise, and biological knowledge required to define models of biological systems.

In many areas of systems biology, this problem has been already solved by designing an intuitive software that can be used by biologists to design and analyze models. For example, there exist excellent software tools for analyzing gene-regulatory or biochemical networks CellDesigner [2], COPASI [3], The Cell Collective [4], and SimBiology [5]. However, in some areas, such as viral infection modelin [6, 7], there is still a possibility to improve existing tools.

There exist several groups of tools dedicated to modeling biological systems that can be applied to model viral infections. Probably the most popular group of tools uses systems of differential equations [8–11]. The application of these tools requires both advanced knowledge and experience with the mathematical analysis. This group of tools is complemented with programs that help to construct models from blocks using graphical user interface, but in this case, the researcher still has to have perfect understanding of how to formally model biological interactions using differential equations. These are programs like Mathworks Simulink [12] and WinFACT [13].

The second group of modeling tools involves designing dedicated formal languages. This group includes, for example, JiST language based on Java [14], SimPy based on Python [15], the immune system simulation frameworks MSI [16], and CAFISS [17]. It also includes XMLlab [18] and SBML [19] languages based on XML which are often used as data storing formats. All of them require the knowledge of some programming language from the user; however, in the case of biologists, it is often not possible to expect them to have even basic programming skills. Furthermore, rule-based approaches [20, 21] that has recently been quite popular fall into this group. They also require the knowledge of formal methods used to describe these rules.

The third group of tools consists of higher level software that has some graphical user interface (GUI) that can be used for easy visual construction of models of biological systems. Currently, this is the most popular group of tools usually extended with the possibility to export or store model description in the SBML format. Unfortunately, all the existing packages either have limited functionality or require preparation of several scripts written in a formal, programming language to supplement the graphically designed part. Example tools in this group are Brahms [22], AndroMeta [23], NetLogo [24], or environments for designing models using SBGN [25] or other notation, the most popular being probably The Cell Collective [4] and SimBiology [5].

The Cell Collective is a tool used to define multiagent models and simulation experiments for system biology. The definition of reaction in this tool is very straightforward; however, it requires to repeat many similar steps a lot of times. It helps users to avoid mistakes by immediate verification of provided reactions at the expense of the overhead required to define complicated simulations with a lot of similar reactions. The graphical interface of SimBiology is quite similar to The Cell Collective; however, it adds the possibility to use MATLAB scripts which can be very helpful provided that the user knows the MATLAB programming language.

All of the above solutions have some disadvantages. They either provide a modeling tool that is not intuitive for a person without advanced mathematical and programming skills or have some functionalities missing. To apply them, the time and cost consuming cooperation with a mathematician or a computer scientist is usually required. This paper presents the description of ModeLang (http://modelang.cs.put.poznan.pl/), the new formal language that can be used to describe viral infection models. It can be a useful tool facilitating cooperation between researchers from different disciplines working on models of viral infections. To make it easy to use, it was designed to be as similar as possible to a natural language [26]. Finally, thanks to the open and free architecture, it can be used as an input for many different modeling techniques [27–30].

The above characteristics of the ModeLang language allow to classify it into the group of Controlled Natural Languages (CNLs). These are languages that look informal like natural languages; however, they have some constraints that allow to process them automatically [31]. It should be stressed that existing CNLs cannot be used instead of ModeLang. Most of them are based on some subset of English that is too general to use to define computer models. These are, for example, ACE [32] that restricts English to the structures that can be interpreted unambiguously, PENG [33] that limits the language a bit more to make parsing easier, and CPL [34] which uses various heuristics to resolve ambiguities. Moreover, all of the mentioned languages translate the input description to the first-order logic which does not help in analyzing them in simulation software.

More helpful could be languages designed for the Semantic Web, which is an attempt to bring structure to data available on the Internet [35]. The goal of these languages is to make it possible to define and query information using a more natural method than formal languages. This goal is very similar to the motivation for creating ModeLang; however, in application for Semantic Web, these languages are used to define ontologies (sets of information) instead of computer models. Most of these languages are based on one of the described CNLs and are related to OWL (Web Ontology Language) which is used to define ontologies [36]. Example languages in this group are ACE View [37], Sydney OWL Syntax [38], Rabbit [39], Lite Natural Language [40], CLOnE [41], or BioPAX [42] that can be used to store biological pathways.

## 2. Materials and Methods

### 2.1. Origins

The idea of ModeLang was drafted as a solution to problems that arised during authors' cooperation with biologists from the Institute of Bioorganic Chemistry in Poland. The final language implementation is a result of over four years of experience in modelling viral infection. At the beginning, the collaboration was very difficult. The turning point occurred after presenting to the biologists rules that described the infection using natural language. Their form was very similar to the one used in ModeLang. The following literature study confirmed that there are reasons [20] to believe that biological modelers who have the ability to understand and modify mathematical models are a minority and that using CNL is a correct solution to that problem [43].

### 2.2. Terminology

ModeLang is the name of the language that was designed to provide a convenient way to create descriptions of models of biological systems. To demonstrate the presented language, two examples from the field of immunology, HCV and HIV infections, are used. Only very basic immunological terms are used to exemplify designed rules and nobody should have problems understanding them. However, if it is the case, any immunology textbook should quickly help (e.g., [44]).

Three main terms are used in this paper to present the ModeLang language design—agents and rules [29]. The term agents refers to all entities (objects) that are basic elements of the biological system modeled, for example, cells or free virions, but also tissues or organisms. The model described in ModeLang may consist of many agent types, each having a different name, which is a regular text that can optionally contain spaces. Interactions between agents that define the model's dynamics are described by rules. For example, the rule can define that healthy cells are infected with virions with some probability and then transform into infected cells. Finally, the term keywords dictionary refers to the database containing knowledge on how various biological words should be interpreted automatically by a computer.

### 2.3. Design Goals

The main goal during the design of the language was to make it easy to use for experts without mathematical background. To achieve this goal, several approaches were used as follows.

1. The ModeLang language is informal. Rules can be written using different words with the same or similar meaning. Passive or active voice can be used. It is also assumed that auxiliary words in model descriptions are useless from the formal point of view so ModeLang tries to skip them.
2. Names of agents are automatically extracted from the rules description so there is no need to define them formally in the description of the model or in the keywords dictionary.
3. The only mathematical part that has to be included is setting values of different parameters. It is done using simple inequalities that define the range of values for parameters.

### 2.4. Rules

**Figure 1:**
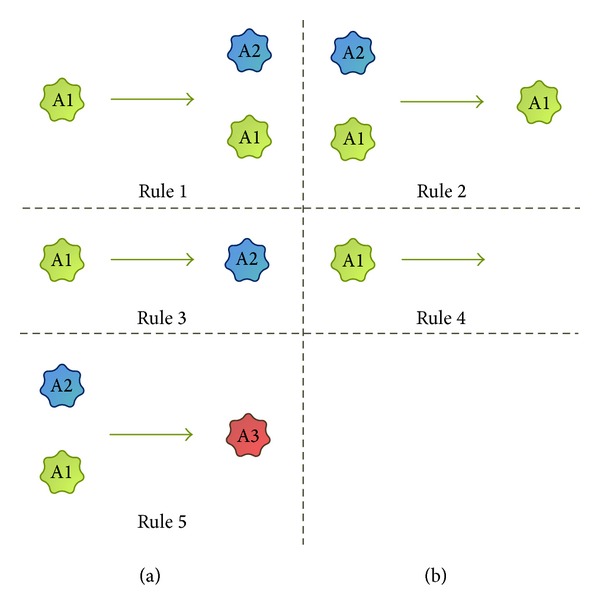
Visualization of rule types. Each item demonstrates the single interaction type between agents. The left part shows the state of the system before execution of the interaction and the right part shows the state after the interaction has taken place.

Based on the analysis of currently used models of viral infection, the following six rules have been identified and defined. Visual demonstration of these rules is presented in Figure 1. In Algorithm 1 there is a short summary of the rules, and below is their more detailed description. The set of rules may seem very limited; however, using the keywords dictionary described later, many different interactions can be modeled using these rules.

*Rule  1: Creation.* This rule describes a process in which one agent creates another agent but it itself remains unchanged. In particular, both agents can be of the same type. For example, a new cell is created by proliferation or the organism is infected with a virus.

*Rule  2: Decay. *This rule describes a process in which one agent destroys another agent. For example, an immune cell can remove a pathogen.

*Rule  3: Transition. *This rule describes a process in which one agent changes into another agent. For example, a new cell is created from its precursor.

*Rule  4: Death. *The agent dies.

*Rule  5: Merge. *This rule describes a process when two agents join to create another agent. For example, a healthy cell can be infected with a virus and can form an infected cell.

*Rule  6: Constraint. *This rule makes it possible to add a constraint on the number of agents. For example, the sum of healthy and infected cells should be smaller than the organism's capacity. In this rule, the word “and” is interpreted as a sum.

### 2.5. Parameters

To make it possible to simulate or analyze the model based on rules, it is required to know how often the interaction described by the rule happens. To define this period, each rule can contain the phrase with parameter defining its frequency. The parameter should be named using any text containing letters and digits without spaces. For each of them, its value has to be defined at the beginning of the model's description. This value can be provided as an equation or a range of values (Algorithm 2). The unit (e.g., milliliter) is skipped but the author of the description should ensure that all units used in it are the same in the whole description.

### 2.6. Keywords Dictionary

It is impossible to automatically interpret rules written in a very flexible natural language without expert knowledge. This knowledge is provided to the automatic interpreter of a model's description through the keywords dictionary. This dictionary should contain all terminology used in the discipline related to the modeled system. Additionally these terms should be assigned to the rule types in which the term can occur. To make the construction of this dictionary easier, it can be written by a computer scientist and a biologist together. When defined, the dictionary can be later used for many different systems from the same field. This paper is accompanied with an example dictionary file that can be used in the viral infection modeling and with a file defining how such dictionaries should be written.

The keywords dictionary should contain the following terms.

1. Verbs and prepositions that are used to formulate each type of rules.
2. Phrases that can be used to introduce parameters to rules.
3. Auxiliary words that can be used for a more flexible formulation of rules (e.g., to use the passive and active voice alternatively).

### 2.7. Implementation

The parser of ModeLang was implemented as a computer program. It reads the models description text, identifies agents, interprets rules, and finds definitions of parameters. Information about each interpreted rule and parameter or potential errors is displayed on the screen to allow biologists to verify that the program understood their intentions correctly.

The output of the program is a computer representation of the described model. To make use of this representation, the algorithm that exports it (hereafter called export algorithm) should be implemented by a computer specialist. The algorithm should convert this representation to the formal input format of the software that is used to analyze the model (e.g., differential equations solver). This step can first seem difficult and it can seem to discourage users from using ModeLang; however, in fact, the most difficult process is parsing the input which is completed by the ModeLang parser. As a result, the export algorithm usually consists of only several instructions for writing the text.

Moreover, the big advantage of ModeLang is its open character. Because of this, the task of implementing export algorithms has to be done only once for each standard that is used for the storage of models. Then, the implemented converters can be easily shared through the ModeLang web page so that all researchers that use this standard can also use ModeLang. This advantage applies to all the software tools described in the introduction.

For example, there exists a large group of tools that can read data stored in SBML format such as Matlab using SBMLToolbox or SimBiology, The Cell Collective, BioNetGen, and many others which extensive review can be found, for example, in [45]. For all of them, it is enough to create a single export algorithm. For all the other tools, it is also easy to implement this algorithm because all of them have well documented input format and as described earlier the export procedure is usually simple.

ModeLang parser is written in C++. It is attached to this paper and can be also downloaded from the language web page (http://modelang.cs.put.poznan.pl/). The computational complexity of the parsing algorithm is polynomial and the parser can process models containing several thousands of rules in less than a second. It can be freely used and modified based on the GNU LGPL license. Keywords dictionary is defined in two XML files and its structure is defined in an XML Schema file. All these files are given in the Supplementary Material available online at http://dx.doi.org/10.1155/2013/320715.

## 3. Results and Discussion

The proposed language and its implementation were tested using the most commonly used models of HCV and HIV infections.

### 3.1. HCV Infection

Based on the model of HCV infection proposed in [46], its description in ModeLang was prepared. The mathematical description of this model consists of the following:

(1) dTdt=s+rTT(1−T+ITmax⁡)−dTT−(1−η)βVT+qI,dIdt=rII(1−T+ITmax⁡)+(1−η)βVT−dII−qI,dVdt=(1−ϵ)pI−cV.

The above mathematical equations are presented to demonstrate how models are usually described. However, thanks to ModeLang, it is no longer required to understand these equations to design a mathematical model. Based on the above model, a friendly ModeLang description was written (see Algorithm 3). Such a description can be created by everyone even without mathematical knowledge and without the need to understand the above equations.

A part of the log that was displayed on the screen during the interpretation of this model to ensure that it was correctly interpreted is presented in Algorithm 4. The rest of the log and the model's description text are given in the Supplementary Material.

**Figure 2:**
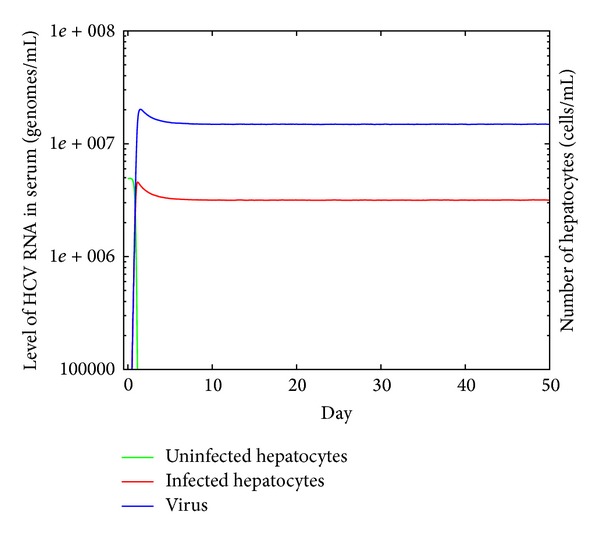
Simulation results of example model described in ModeLang. The graph presents the number of infected hepatocytes, uninfected hepatocytes, and free virions according to the simulation of the HCV infection model defined in ModeLang. The parser prepared during this research was integrated with simulation software and used to read the description of the model prepared by computer scientists together with biologists.

Finally, the constructed computer representation of the model was integrated with the viral infection simulation software developed by the authors [29] to prove that it can be used in practice. Results of this simulation are presented in Figure 2.

### 3.2. HIV Infection

Based on the model of HIV infection proposed in [47], its description in ModeLang was prepared. The mathematical description of this model consists of the following:

(2) $$\begin{array}{c} \frac{dT}{dt}=s-\mu_{T}T+rT\left(1-\frac{T+I}{T_{\max}}\right)-k_{1}VT,\\ \frac{dI}{dt}=k_{1}VT-\mu_{I}I,\\ \frac{dV}{dt}=N\mu_{B}I-k_{1}VT-\mu_{V}V. \end{array}$$

The ModeLang description that was written based on this model is in Algorithm 5.

The description was successfully interpreted by the ModeLang parser. The rest of the log and the model's description text are given in the Supplementary Material.

## 4. Conclusions

ModeLang is an innovative, experts-friendly language for creating viral infection models description. The results presented by authors clearly show that the description written in ModeLang is much easier to understand, modify, and create from scratch for biologists than a definition of models based on differential equations. For models containing many similar reactions, it is much easier to define them in ModeLang than in GUI applications like The Cell Collective because the text can be easily copied and adjusted. Also, creating a new model based on the old one is a very simple process if it contains many similar reactions. In GUI-based tools, it is a serious problem, while in ModeLang, it is easy to copy the text describing similar reactions to the new file. Another advantage of ModeLang based on the controlled natural languages approach is the self-documentation. The rules are presented in an easy to interpret format, similar to a natural language so everyone can easily understand them even without the knowledge of ModeLang. Although such models have not been presented in this paper due to space limitations, it was computationally verified that ModeLang can handle models consisting of thousands of interactions.

The reason for defining programming languages dedicated to describing models and simulations is to make it possible to easily interpret them by a computer. However, at the same time, it makes them difficult to interpret by users without programming skills. The ModeLang solves this problem by providing a language that is both formal and intuitive. It can be parsed by a computer and quickly converted to any other required format. This format can be for example, SBML but also the format of some new application published recently in the Internet that can facilitate the research work of scientists who already have some models defined in ModeLang. It can be also used and modified by every type of user (e.g., biologist) in a straightforward way.

As a result, the use of ModeLang can significantly speed up computational verification of formulated biological hypotheses. This will make the process of designing new therapies and medicines much easier, faster, and cheaper, and as a result, it can save many lives and improve the health of many patients.

To make sure that ModeLang is biologists-friendly, it was consulted with biologists from the Institute of Bioorganic Chemistry in Poznan during the research phase. This cooperation helped to improve ModeLang and to make it easier to use. ModeLang can definitely be an interesting tool for all experts that analyze viral infections and it can become an important bridge between biologists and mathematicians specializing in a modeling software. Additionally, after preparation of suitable keywords dictionaries, it can be successfully used in other disciplines.

## Supplementary Material

The Supplementary Material for this article containts four file. (1) Example XML files packed into one ZIP archive with the definition of keywords used to describe viral infection models. (2) The XML Schema (XSD) definition of the format that should be used to create an XML file with a keywords dictionary. (3) The source code and the Windows binary of an example parser implementation written in C++. (4) Archive with definition of example models of HIV and HCV infection presented in the article together with a full log from parsing process.

## Figures and Tables

**Algorithm 1:**
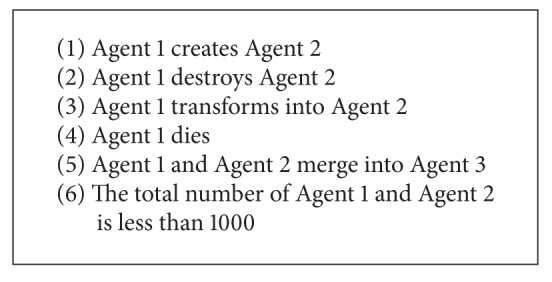


**Algorithm 2:**
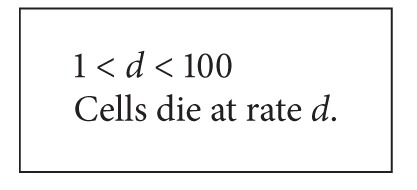


**Algorithm 3:**
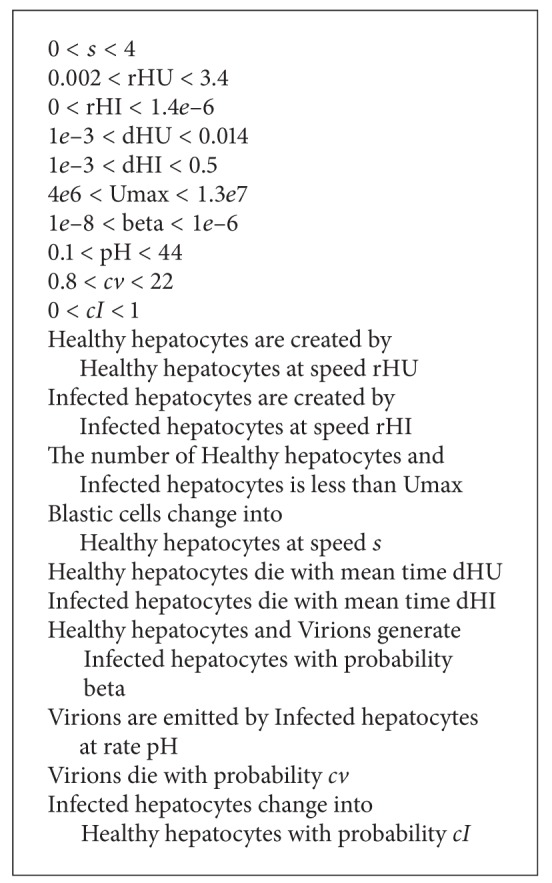


**Algorithm 4:**
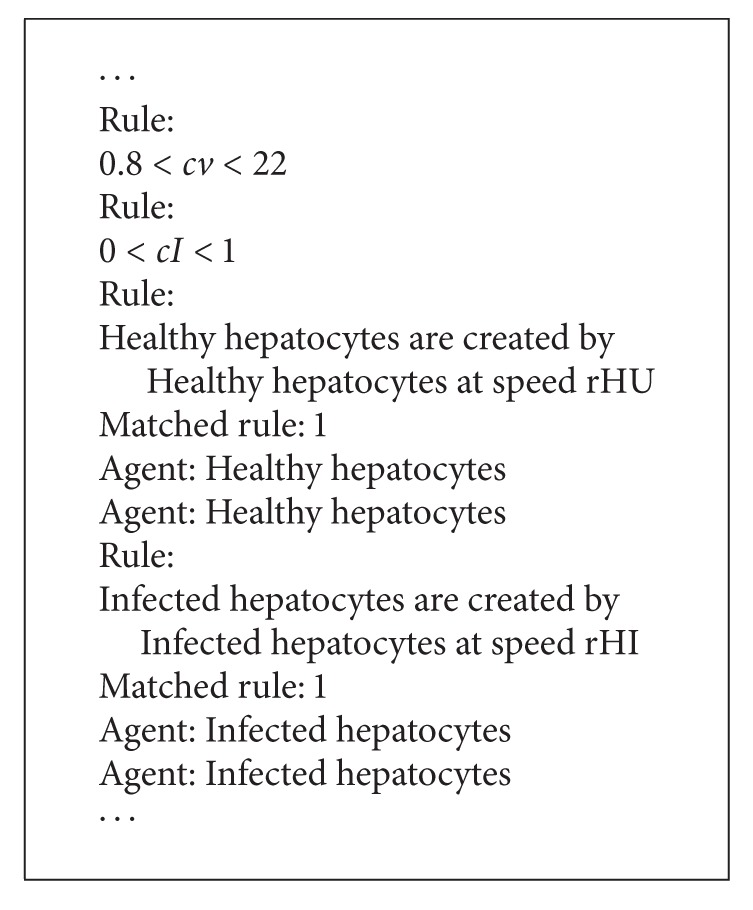


**Algorithm 5:**